# Combined pelvic floor training and hormonal therapy for endometriosis pain recurrence: a propensity score-matched study

**DOI:** 10.3389/fmed.2026.1850107

**Published:** 2026-08-31

**Authors:** Jie Song, Hui Dong, Zhen Chen

**Affiliations:** 1 Department of Gynecology, Tianjin Central Hospital of Gynecology Obstetrics, Tianjin, China; 2 Tianjin Medical University, Tianjin, China; 3 School of Medicine, Nankai University, Tianjin, China; 4 Department of Obstetrics, Tianjin Central Hospital of Gynecology Obstetrics, Tianjin, China; 5 Tianjin Key Laboratory of Human Development and Reproductive Regulation, Tianjin, China

**Keywords:** combined oral contraceptives, EHP-30, endometriosis, long-term, pain recurrence, pelvic floor muscle training, propensity score matching, quality of life

## Abstract

**Background:**

Endometriosis (EMS) causes debilitating pain and reduces quality of life. Whether pelvic floor muscle training (PFMT) provides independent, additive benefits beyond standard postoperative hormonal suppression remains unknown.

**Methods:**

A retrospective cohort study was conducted on 446 women with surgically confirmed EMS between June 2022 and May 2024. Patients were categorized into five groups: PFMT-only (*n* = 98), PFMT+long-term adjuvant progestogen (PFMT+LAP; *n* = 91), PFMT+combined oral contraceptives (PFMT+COC; *n* = 87), LAP-only (*n* = 85), and COC-only (*n* = 85). Propensity score matching (PSM) with 28 baseline covariates was applied to isolate the effects of PFMT. Primary outcomes included 12-month pain recurrence, health-related quality of life (EHP-30), and recurrence-free survival.

**Results:**

After PSM, 12-month dysmenorrhea recurrence was significantly lower in combination groups compared to hormonal therapy alone: PFMT+LAP vs. LAP-only (36.5% vs. 75.0%; OR = 0.192; *p* = 0.0002) and PFMT+COC vs. COC-only (38.3% vs. 68.1%; OR = 0.291; *p* = 0.0072). Multivariable logistic regression confirmed PFMT as an independent protective factor against recurrence for dysmenorrhea, chronic pelvic pain, and dyspareunia (all p<0.005). No significant difference was found between LAP-only and COC-only groups (OR = 1.04; *p* = 0.914), indicating PFMT drove the incremental benefit. Linear mixed-effects modeling showed a significant time-group interaction for EHP-30 pain scores (*F* = 39.14; p<0.001). Kaplan-Meier analysis demonstrated superior recurrence-free survival for combination regimens (log-rank p<0.001).

**Conclusion:**

Pelvic floor muscle training combined with hormonal therapy significantly reduces pain recurrence and quality-of-life burden while extending recurrence-free survival compared to hormonal therapy alone. These findings suggest that PFMT may confer a clinically meaningful adjunctive benefit. However, given the retrospective, non-randomized design of this study, confirmation in prospective randomized controlled trials is warranted before routine integration into postoperative endometriosis management protocols can be recommended.

## 1 Introduction

Endometriosis (EMS) is a chronic, estrogen-dependent inflammatory disease characterized by the presence of endometrial-like tissue outside the uterus, most commonly affecting women of reproductive age (1). It is estimated to affect 6%–10% of women worldwide, with higher prevalence among those with chronic pelvic pain or infertility (2). The cardinal symptoms — dysmenorrhea, chronic pelvic pain (CPP), dyspareunia, and infertility — impose a substantial burden on health-related quality of life and socioeconomic productivity (3). Despite advances in surgical and medical management, endometriosis remains a recurrent condition, with pain recurrence rates following conservative surgery ranging from 20% to 40% within 2 years (4).

Progestogens such as dienogest and combined oral contraceptives (COCs) represent mainstay postoperative hormonal therapies, both demonstrating efficacy in reducing recurrence risk and ameliorating associated pain (5, 6). Nevertheless, a significant proportion of women experience persistent symptoms despite optimal medical therapy, highlighting the need for adjunctive, non-pharmacological approaches (7).

Recent attention has focused on pelvic floor dysfunction as a key contributor to endometriosis-related pain (7, 8). The pathogenesis of endometriosis involves complex interactions among immune dysregulation, neuroangiogenesis, and musculoskeletal sensitization (8, 9). Chronic pain states may induce hypertonicity and impaired coordination of the pelvic floor musculature, thereby amplifying symptoms including dysmenorrhea and dyspareunia (9). Pelvic floor muscle training (PFMT) targets this neuromuscular dysfunction and has demonstrated benefit in reducing chronic pelvic pain and improving quality of life in women with endometriosis (10). However, rigorous data on PFMT as part of a multimodal postoperative regimen remain limited (11).

A critical methodological gap is that no study has rigorously evaluated whether PFMT confers benefit specifically beyond hormonal therapy alone, a comparison requiring dedicated hormone-only control groups (12, 13). The present study was designed to address this gap using a five-group design incorporating two hormone-only control cohorts, with propensity score matching to isolate the independent contribution of PFMT.

## 2 Materials and methods

### 2.1 Inclusion and exclusion criteria

Inclusion criteria: (1) Surgically confirmed endometriosis (14); (2) Underwent laparoscopic or open surgery to remove visible endometriotic lesions; (3) Female patients aged 18–45 years; (4) r-ASRM stage I–IV (15); (5) Significant EMS-related pain symptoms (dysmenorrhea, CPP, or dyspareunia); (6) Complete medical records without missing data.

Exclusion criteria: (1) Pregnancy or lactation; (2) Severe hepatic, renal, or cardiac disease, or uncontrolled diabetes; (3) Allergy to study medications; (4) Receipt of hormone therapy or PFMT within the previous 6 months; (5) Uncontrolled psychiatric disorder; (6) Planning pregnancy within the next year; (7) Postoperative infection or severe bleeding.

### 2.2 Study design and data collection

A retrospective cohort analysis was conducted on 446 patients with surgically confirmed EMS treated at our institution between June 2022 and May 2024. To directly address whether PFMT confers additive benefit beyond hormonal therapy alone, two additional retrospective control groups were identified from the same institutional database during the same period under identical eligibility criteria: patients receiving dienogest alone without concurrent PFMT (LAP-only; *n* = 85) and patients receiving COC alone without PFMT (COC-only; *n* = 85). The final cohort comprised five groups: PFMT alone (*n* = 98), PFMT+LAP (*n* = 91), PFMT+COC (*n* = 87), LAP-only (*n* = 85), and COC-only (*n* = 85). All patients were followed for 12 months post-surgery with scheduled assessments at baseline, 3 months, and 6 months. The historical control groups were identified from institutional records preceding the formal incorporation of physiotherapist-supervised PFMT into the postoperative management pathway. Institutional protocol updates introduced structured PFMT as a standard adjunct to hormonal therapy during the study period; patients enrolled prior to this revision therefore received hormonal therapy alone, reflecting the prevailing standard of care at that time rather than patient preference or resource constraints.

### 2.3 Postoperative management

All PFMT-containing groups commenced PFMT on the day of discharge: three 15-min sessions per week for 6 months. PFMT was delivered under the direct supervision of a certified physiotherapist using a dedicated pelvic floor rehabilitation device, comprising three sequential components: (1) low-frequency electrical stimulation targeting pelvic floor muscle relaxation; (2) surface electromyography (sEMG)-guided biofeedback for voluntary relaxation training; and (3) therapist-directed pelvic floor elongation and relaxation exercises. The protocol was designed as a neuromuscular downtraining regimen, consistent with the hypertonic pelvic floor dysfunction commonly observed in endometriosis-associated chronic pelvic pain. The PFMT+LAP group additionally received oral dienogest 2 mg daily for 6 months. The PFMT+COC group received daily drospirenone 3 mg/ethinyl estradiol 0.03 mg (Yasmin, Bayer, Germany) for 6 months. The LAP-only and COC-only groups received respective hormonal therapies without PFMT, following the same drug protocols. Treatment adherence was confirmed for all enrolled participants across all groups through institutional medical records review. All patients completed the full course of their assigned postoperative treatment without documented interruption.

### 2.4 Outcome measures

Pain severity was assessed using the Visual Analogue Scale (VAS; 0–10 cm) at baseline, 3 months, and 6 months (16). Health-related quality of life was measured using the Endometriosis Health Profile-30 (EHP-30), the validated disease-specific instrument recommended by the World Endometriosis Research Foundation EPHect guidelines (17). The EHP-30 comprises five core subscales (pain, control and powerlessness, emotional well-being, social support, and self-image); each is scored 0–100, with higher scores indicating worse quality of life. Assessments were performed at baseline, 3 months, and 6 months. The Short Form Health Survey-12 (SF-12) Physical Component Summary (PCS) and Mental Component Summary (MCS) scores were additionally recorded to enable comparison with broader pain literature (18). Twelve-month cumulative pain recurrence (dysmenorrhea, CPP, and dyspareunia) and time to first recurrence were the primary 12-month endpoints.

### 2.5 Ethics statement

The study was approved by the Medical Ethics Committee of Tianjin Central Hospital of Gynecology and Obstetrics (Ethics Reference Number: 2025KY090). This study was conducted in accordance with the Declaration of Helsinki. The requirement for informed consent was waived by the Medical Ethics Committee of Tianjin Central Hospital of Gynecology and Obstetrics due to the retrospective nature of the study.

### 2.6 Statistical analysis

Statistical analyses were performed using R version 4.3 (R Core Team, 2023). Given the non-randomized retrospective design, PSM was the primary analytic strategy to mitigate selection bias. Propensity scores were estimated via logistic regression incorporating 28 pre-specified baseline covariates: age, BMI, CA-125, r-ASRM stage, surgical approach, baseline pain symptom status (dysmenorrhea, CPP, dyspareunia), pre-treatment VAS scores for each symptom, SF-12 PCS and MCS, prior NSAID use, educational level, marital status, menstrual regularity, gravidity, infertility history, alcohol and tobacco use, ectopic lesion location, menarchal age, menstrual cycle length, duration of menses, and intraoperative findings (resection completeness, deep infiltrating EMS, adhesion score). Nearest-neighbor 1:1 matching with a caliper of 0.3 standard deviations was applied separately for PFMT+LAP vs. LAP-only and PFMT+COC vs. COC-only using the MatchIt package (19). Covariate balance was assessed by standardized mean differences (SMD; target <0.10) and Love plots.

For PSM binary recurrence outcomes, chi-square tests with Yates’ continuity correction were used, and results were expressed as ORs with 95% CIs. Full-cohort multivariable logistic and linear regression analyses (reference: LAP-only) served as sensitivity analyses. Repeated EHP-30 measurements (baseline, 3 months, 6 months) were modeled with linear mixed-effects models with fixed effects for time, group, and their interaction, and a random intercept per subject [lme4 and lmerTest packages (20)]; estimated marginal means were derived using emmeans (21). The EHP-30 pain subscale was designated the primary quality-of-life domain; analogous models for the emotional well-being and control subscales showed consistent results (emotional well-being: *F* = 39.18, *p* < 2 × 10^−16^; total score: *F* = 39.05, *p* < 2 × 10^−16^). Twelve-month recurrence-free survival was estimated by the Kaplan–Meier method; pairwise log-rank tests with Bonferroni correction evaluated between-group differences. Multivariable Cox proportional hazards models, adjusted for the same 28 covariates, estimated hazard ratios (HRs); the proportional hazards assumption was evaluated using Schoenfeld residuals. Post-hoc power analysis used Cohen’s *h* for two proportions (pwr package) (22). All tests were two-sided with α = 0.05.

## 3 Results

### 3.1 Baseline characteristics

Baseline demographic and clinical characteristics across the five groups are presented in Tables 1, 2. The five groups were broadly comparable on most baseline variables. The surgical approach did not differ significantly across groups (laparoscopy: PFMT 70.4%, PFMT+LAP 69.2%, PFMT+COC 74.7%, LAP-only 63.5%, COC-only 62.4%; *p* = 0.385), reflecting adequate comparability between original and added control groups. Age at menarche was modestly different across groups (*p* < 0.001; SMD = 0.336), driven by a slight difference between PFMT-containing and hormone-only cohorts; this variable was incorporated as a covariate in all PSM and regression models. Duration of menses (*p* = 0.770; SMD = 0.088), CA-125 (*p* = 0.475), and all baseline pain measures were not significantly different across groups (all *p* > 0.05), confirming overall baseline comparability.

**TABLE 1:** Demographic characteristics across five treatment groups.

Parameter	PFMT (*n* = 98)	PFMT+LAP (*n* = 91)	PFMT+COC (*n* = 87)	LAP-only (*n* = 85)	COC-only (*n* = 85)	*p*
Age (years, mean ± SD)	28.86 ± 2.22	29.29 ± 2.72	28.64 ± 2.76	29.08 ± 2.99	29.06 ± 2.91	0.580
BMI (kg/m^2^, mean ± SD)	20.29 ± 2.36	20.32 ± 2.47	20.85 ± 2.37	20.91 ± 2.21	20.54 ± 2.38	0.240
Age of menarche (years)	12.25 ± 1.46	12.13 ± 1.36	12.14 ± 1.52	12.74 ± 0.77	12.79 ± 0.68	<0.001
Menstrual cycle length (days)	30.09 ± 2.10	29.52 ± 2.54	29.64 ± 2.77	29.21 ± 3.17	29.73 ± 2.95	0.277
Duration of menses (days)	4.57 ± 0.91	4.53 ± 1.25	4.49 ± 1.04	4.59 ± 0.97	4.69 ± 1.05	0.770
CA-125 (U/mL, mean ± SD)	78.62 ± 21.53	78.36 ± 21.44	79.84 ± 20.75	83.73 ± 21.51	80.78 ± 23.10	0.475
Gravidity = 1 [*n* (%)]	52 (53.1%)	50 (54.9%)	38 (43.7%)	49 (57.6%)	47 (55.3%)	0.394
Infertility history [*n* (%)]	19 (19.4%)	18 (19.8%)	20 (23.0%)	18 (21.2%)	16 (18.8%)	0.963

PFMT, pelvic floor muscle training; LAP, long-term adjuvant progestogen; COC, combined oral contraceptives; SD, standard deviation.

**TABLE 2:** Disease features and surgical characteristics across five groups.

Parameter	PFMT (*n* = 98)	PFMT+LAP (*n* = 91)	PFMT+COC (*n* = 87)	LAP-only (*n* = 85)	COC-only (*n* = 85)	*p*
Ectopic location: Ovary [*n* (%)]	64 (65.3%)	59 (64.8%)	60 (69.0%)	53 (62.4%)	48 (56.5%)	0.856
r-ASRM stage I–II [*n* (%)]	78 (79.6%)	72 (79.2%)	69 (79.3%)	69 (81.2%)	67 (78.8%)	1.000
r-ASRM stage III–IV [*n* (%)]	20 (20.4%)	19 (20.9%)	18 (20.7%)	16 (18.8%)	18 (21.2%)	–
Laparoscopy [*n* (%)]	69 (70.4%)	63 (69.2%)	65 (74.7%)	54 (63.5%)	53 (62.4%)	0.385
Dysmenorrhea at baseline [*n* (%)]	72 (73.5%)	66 (72.5%)	68 (78.2%)	66 (77.6%)	55 (64.7%)	0.276
CPP at baseline [*n* (%)]	53 (54.1%)	49 (53.8%)	45 (51.7%)	53 (62.4%)	54 (63.5%)	0.384
Dyspareunia at baseline [*n* (%)]	60 (61.2%)	51 (56.0%)	57 (65.5%)	61 (71.8%)	62 (72.9%)	0.095
NSAIDs use at baseline [*n* (%)]	85 (86.7%)	80 (87.9%)	75 (86.2%)	68 (80.0%)	75 (88.2%)	0.527

r-ASRM, revised American Society for Reproductive Medicine staging. Stages I–II and III–IV were combined for reporting purposes due to the small number of stage III–IV cases per group (range 18.8%–21.2%); *p*-values reflect the overall four-level chi-square test. CPP, chronic pelvic pain; NSAIDs, non-steroidal anti-inflammatory drugs.

### 3.2 Propensity score matching and primary comparative analysis

**FIGURE 1:**
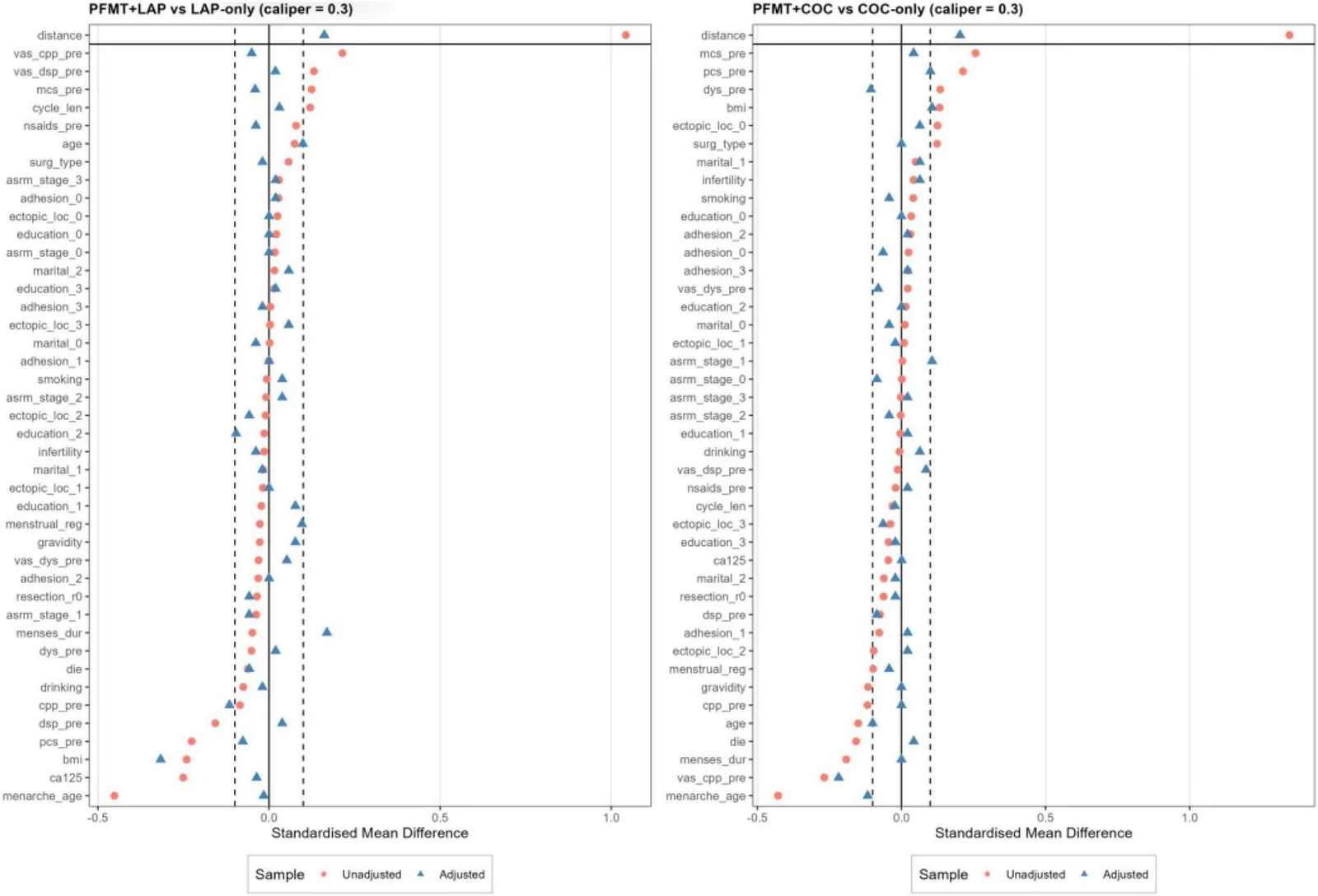
Covariate balance before and after propensity score matching. Left: PFMT+LAP vs. LAP-only (52 matched pairs). Right: PFMT+COC vs. COC-only (47 matched pairs). Salmon circles represent pre-matching standardized mean differences (SMD); blue triangles represent post-matching SMD. Dashed vertical lines denote the SMD = ± 0.10 threshold.

After PSM (caliper = 0.3 SD), 52 matched pairs were retained for the PFMT+LAP vs. LAP-only comparison and 47 matched pairs for PFMT+COC vs. COC-only. Post-matching covariate balance was satisfactory, with most pairwise SMDs below 0.20 after matching, as illustrated in Figure 1.

**FIGURE 2:**
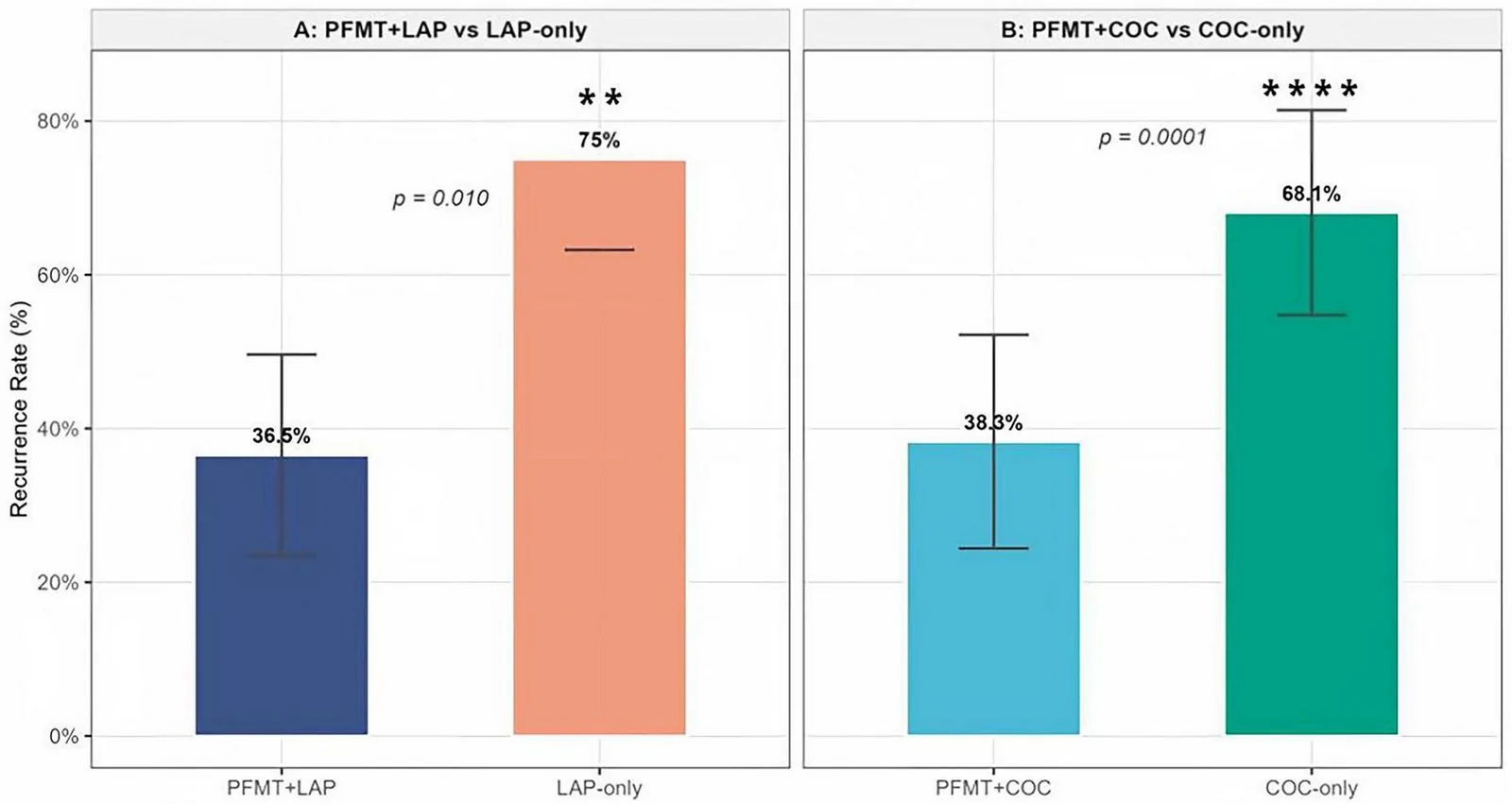
Twelve-month dysmenorrhea recurrence rates in PSM-matched cohorts. **(A)** PFMT+LAP vs. LAP-only (*n* = 52 matched pairs); ****, *p* < 0.0001 (*p* = 0.0002). **(B)** PFMT+COC vs. COC-only (*n* = 47 matched pairs); **, *p* < 0.01 (*p* = 0.007). Bars represent 12-month recurrence rates; error bars indicate 95% confidence intervals.

In the matched PFMT+LAP vs. LAP-only cohort, 12-month dysmenorrhea recurrence was significantly lower in PFMT+LAP (36.5% vs. 75.0%; OR = 0.192; 95% CI 0.077–0.480; *p* = 0.0002). The higher recurrence rate observed in the PSM-matched LAP-only subgroup (75.0%) compared with the full LAP-only cohort (64.7%) reflects selection of higher-risk patients during nearest-neighbor matching, a known feature of PSM algorithms. CPP recurrence was also significantly reduced (23.1% vs. 46.2%; OR = 0.350; *p* = 0.023). In the matched PFMT+COC vs. COC-only cohort, dysmenorrhea recurrence was markedly lower in PFMT+COC (38.3% vs. 68.1%; OR = 0.291; 95% CI 0.105–0.804; *p* = 0.0072). These findings are displayed in Figure 2.

Continuous 6-month outcomes were also consistently superior in combination groups within matched cohorts. EHP-30 total scores were significantly lower for PFMT+LAP vs. LAP-only (31.25 vs. 39.17; *p* = 0.004) and PFMT+COC vs. COC-only (31.42 vs. 41.06; *p* = 0.001). MCS scores were markedly higher in both combination groups (PFMT+LAP vs. LAP-only: 97.65 vs. 88.12; *p* < 0.001; PFMT+COC vs. COC-only: 98.05 vs. 90.06; *p* < 0.001).

Full-cohort multivariable logistic regression (reference: LAP-only) corroborated the PSM results (Table 3). PFMT+LAP (OR = 0.305; 95% CI 0.154–0.594; *p* = 0.0006) and PFMT+COC (OR = 0.270; 95% CI 0.134–0.532; *p* = 0.0002) both demonstrated substantially reduced odds of 12-month dysmenorrhea recurrence. CPP recurrence was also significantly reduced by PFMT+LAP (OR = 0.321; *p* = 0.002) and PFMT+COC (OR = 0.278; *p* < 0.001), as was dyspareunia recurrence (PFMT+LAP OR = 0.298; *p* = 0.001; PFMT+COC OR = 0.253; *p* < 0.001). The COC-only vs. LAP-only comparison did not reach statistical significance for any pain outcome (dysmenorrhea OR = 1.04; *p* = 0.914), confirming that PFMT — not the specific hormonal agent — is the active incremental component of the combined regimen.

**TABLE 3:** Multivariable logistic and linear regression results for 12-month outcomes (reference: LAP-only; *n* = 446).

Outcome	Group	OR or β	95% CI	*p*
Dysmenorrhea recurrence	PFMT	0.518	0.267–0.990	0.048
	PFMT+LAP	0.305	0.154–0.594	<0.001
	PFMT+COC	0.270	0.134–0.532	<0.001
	COC-only	1.040	0.527–2.050	0.914
CPP recurrence	PFMT	0.751	0.391–1.440	0.386
	PFMT+LAP	0.321	0.156–0.647	0.002
	PFMT+COC	0.278	0.131–0.573	<0.001
	COC-only	0.968	0.505–1.860	0.922
Dyspareunia recurrence	PFMT	0.699	0.356–1.370	0.296
	PFMT+LAP	0.298	0.139–0.619	0.001
	PFMT+COC	0.253	0.116–0.535	<0.001
	COC-only	1.110	0.565–2.190	0.758
EHP-30 total score (β)	PFMT+LAP	−7.52	−9.98 to −5.06	<0.001
	PFMT+COC	−9.54	−12.0 to −7.04	<0.001
VAS dysmenorrhea (β)	PFMT+LAP	−0.463	−0.722 to −0.203	<0.001
	PFMT+COC	−0.539	−0.803 to −0.276	<0.001

OR, odds ratio; β, regression coefficient; CI, confidence interval. Adjusted for all 28 baseline covariates. PFMT-alone and COC-only rows are included for completeness; the COC-only vs. LAP-only comparison did not reach statistical significance for any pain outcome. CPP, chronic pelvic pain; EHP-30, Endometriosis Health Profile-30; VAS, visual analogue scale.

*Post-hoc* power analysis yielded Cohen’s h of 0.531 (PFMT+LAP vs. LAP-only) and 0.590 (PFMT+COC vs. COC-only). Post-hoc power at the PSM-matched cohort sizes was 0.773 (*n* = 52 pairs) and 0.816 (*n* = 47 pairs), respectively; at the full unmatched cohort size (*n* = 85 per group), power exceeded 0.934. Minimum sample sizes required for 80% power were 56 and 46 per group, respectively.

### 3.3 EHP-30 quality of life: linear mixed-effects model

**FIGURE 3:**
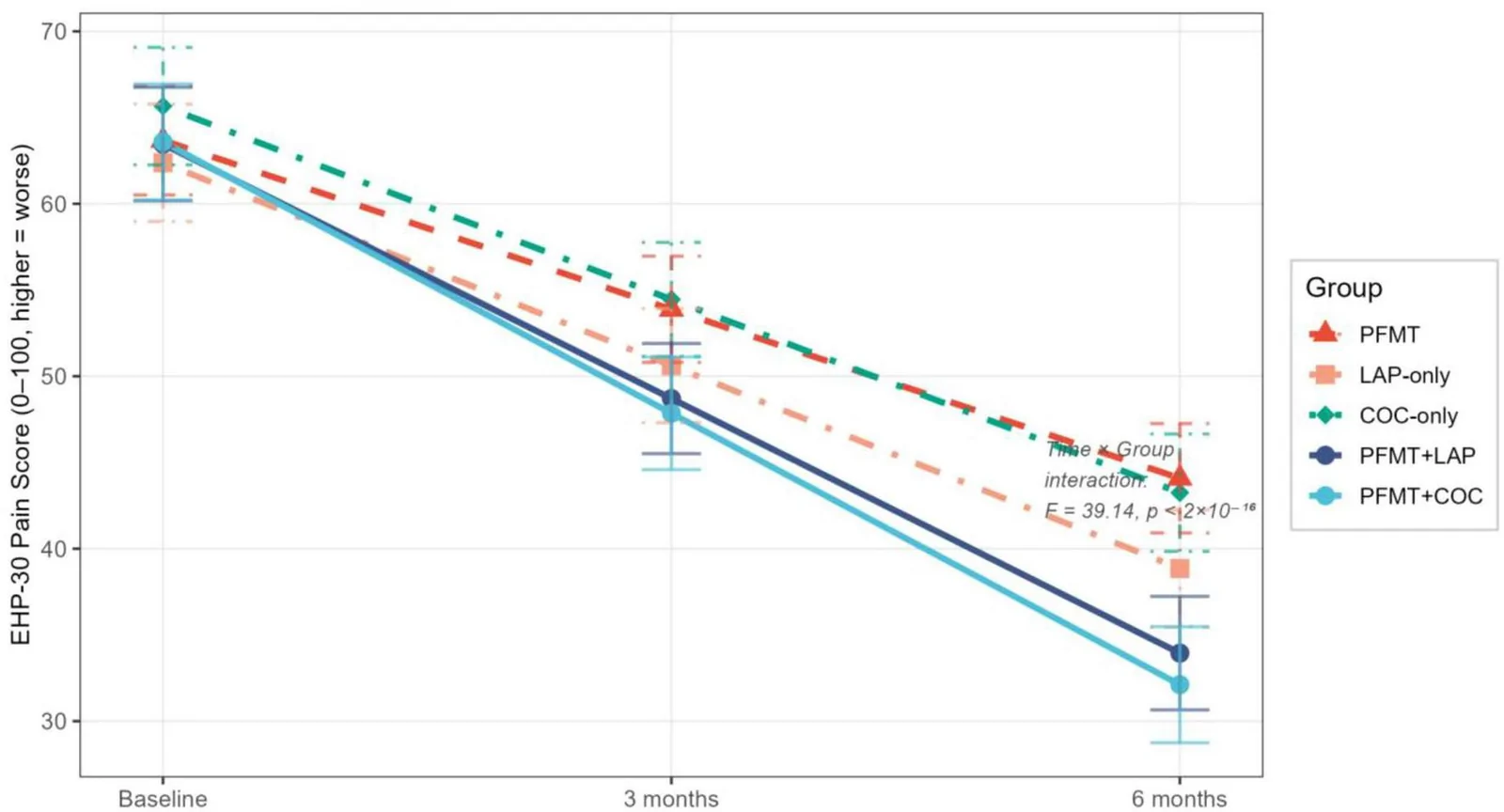
Endometriosis Health Profile-30 (EHP-30) pain subscale: estimated marginal means over time by treatment group. Points represent estimated marginal means derived from linear mixed-effects models. Error bars indicate 95% confidence intervals. Solid lines represent PFMT-containing combination therapy groups; dashed lines represent hormone-only and PFMT-alone groups. Time × group interaction: *F* = 39.14, df = 4/892, *p* < 2 × 10^−16^. Higher scores indicate worse quality of life (scale 0–100).

Linear mixed-effects modeling of EHP-30 pain subscale scores revealed a highly significant time × group interaction (*F* = 39.14; df = 4/892; *p* < 2 × 10^−16^), indicating that improvement trajectories differed systematically among groups (Figure 3). The main effect of group was non-significant (*F* = 0.48; *p* = 0.753), confirming that differences arose from differential rates of improvement over time rather than pre-existing baseline disparities. Analogous interactions were significant for the EHP-30 total score (*F* = 39.05; *p* < 2 × 10^−16^) and emotional well-being subscale (*F* = 39.18; *p* < 2 × 10^−16^), confirming a consistent pattern across all EHP-30 dimensions.

At baseline, estimated marginal mean EHP-30 pain scores were comparable across all five groups (range 62.4–65.7; all pairwise *p* > 0.05). By 6 months, PFMT+LAP (33.9; 95% CI 30.7–37.2) and PFMT+COC (32.1; 95% CI 28.7–35.5) were significantly lower than LAP-only (38.9; 95% CI 35.4–42.3), COC-only (43.2; 95% CI 39.8–46.7), and PFMT alone (44.1; 95% CI 40.9–47.3).

### 3.4 EMS-related pain recurrence at 3 and 6 months

The following analyses are based on the original three PFMT-containing groups with 6-month follow-up data, complementing the 12-month PSM primary analysis in section “3.2 Propensity score matching and primary comparative analysis.” At 6 months, dysmenorrhea recurrence was 42.86% (PFMT), 27.47% (PFMT+LAP), and 25.29% (PFMT+COC; both *p* < 0.05 vs. PFMT). Similar patterns were observed for CPP (PFMT: 22.45%; PFMT+LAP: 10.99%; PFMT+COC: 10.34%) and dyspareunia (PFMT: 41.84%; PFMT+LAP: 25.27%; PFMT+COC: 26.44%; all *p* < 0.05 vs. PFMT). These 6-month rates are each lower than the corresponding 12-month rates in section “3.2 Propensity score matching and primary comparative analysis,” consistent with the natural history of progressive pain recurrence over extended follow-up.

### 3.5 VAS pain scores

Baseline VAS scores were comparable across all five groups (all *p* > 0.05). At 6 months, multivariable regression (reference: LAP-only) confirmed significantly lower VAS dysmenorrhea for PFMT+LAP (β = −0.463; *p* < 0.001) and PFMT+COC (β = −0.539; *p* < 0.001), while COC-only was not significant (*p* = 0.496). Within matched cohorts, VAS dysmenorrhea was lower for PFMT+LAP vs. LAP-only (1.82 vs. 2.33; *p* = 0.009; PSM-matched cohort, two-sample *t*-test) with a consistent trend for PFMT+COC vs. COC-only (1.79 vs. 2.16; *p* = 0.086; PSM-matched cohort, two-sample *t*-test).

### 3.6 NSAIDs use

**FIGURE 4:**
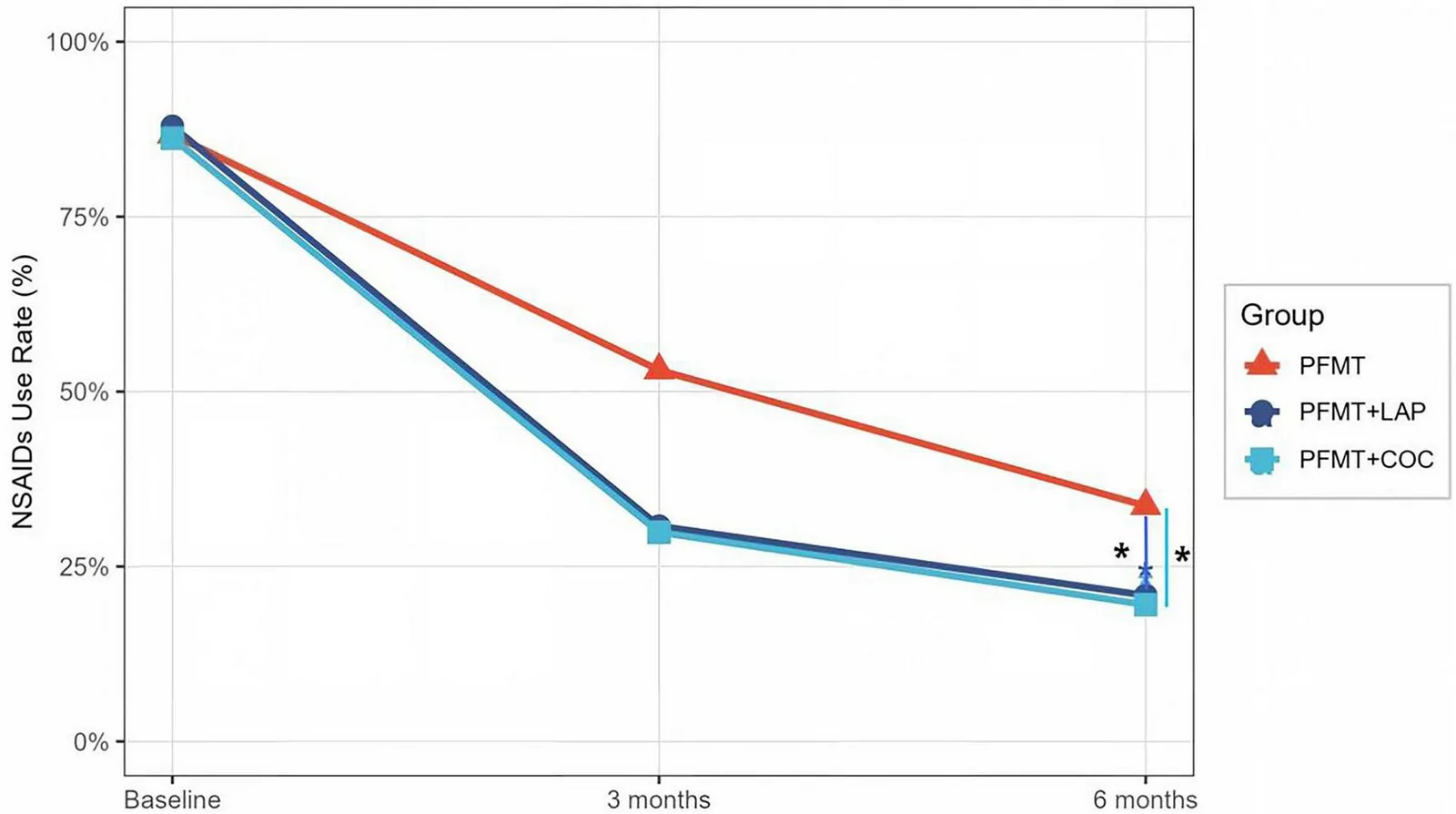
Non-steroidal anti-inflammatory drugs (NSAIDs) use rate among the three PFMT-containing groups at baseline, 3 months, and 6 months. *Data presented as proportions of patients using non-steroidal anti-inflammatory drugs at each time point, *p* < 0.05 compared with PFMT-alone group. Unadjusted comparison within the original three cohorts.

At 6 months, NSAID use was significantly lower in both combination groups versus PFMT alone: 20.88% (PFMT+LAP) and 19.54% (PFMT+COC) versus 33.67% (PFMT; both *p* < 0.05; Figure 4).

### 3.7 Kaplan–Meier recurrence-free survival

**FIGURE 5:**
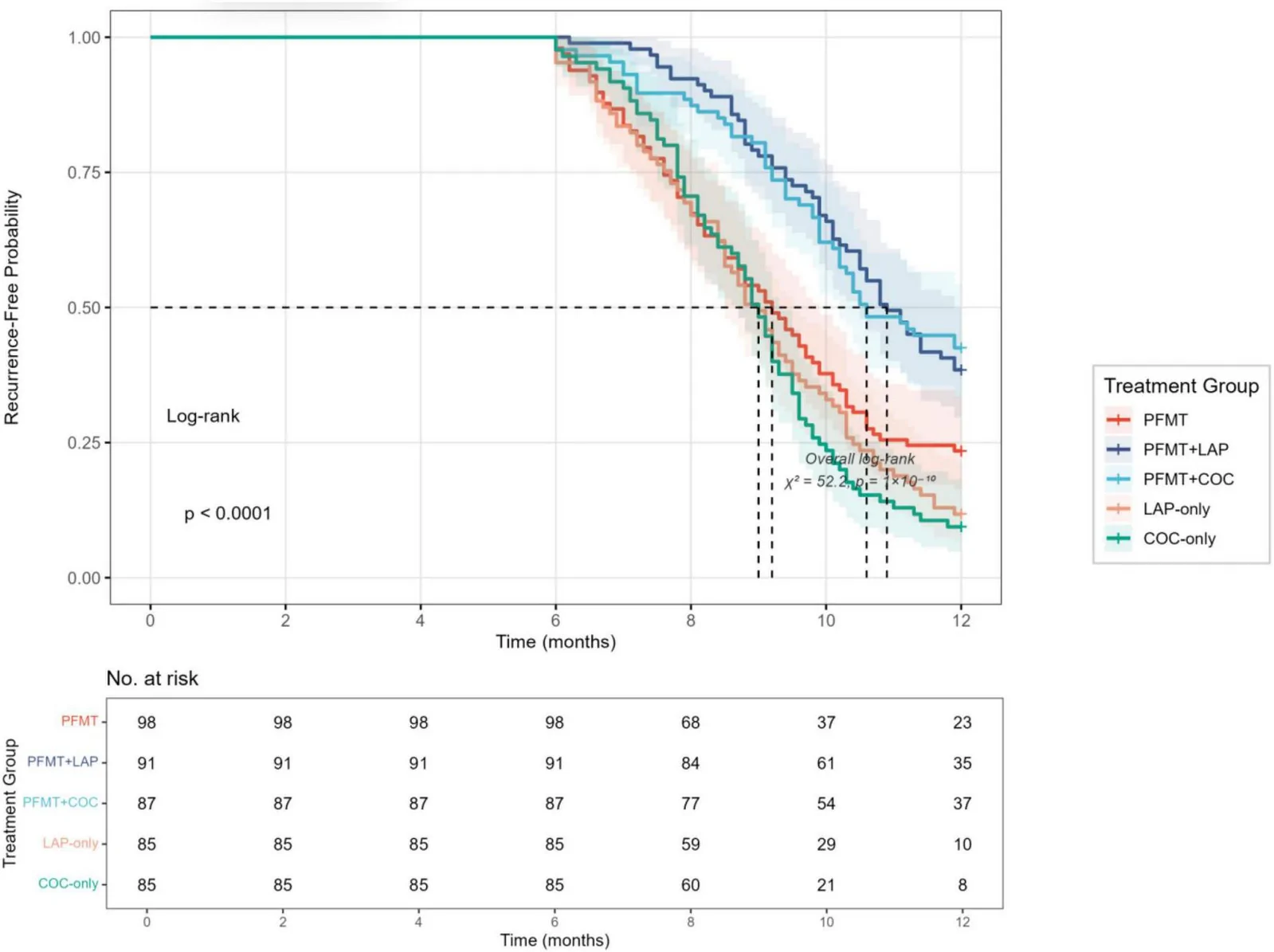
Kaplan–Meier recurrence-free survival curves for all five treatment groups over 12 months. Tick marks indicate censored observations; numbers at risk are shown below. Overall log-rank: χ^2^ = 58.5, *p* = 6 × 10^–12^. Pairwise Bonferroni-corrected comparisons: PFMT+LAP vs. LAP-only, *p* = 1.5 × 10^–6^; PFMT+COC vs. COC-only, *p* = 6.4 × 10^–8^; LAP-only vs. COC-only, *p* = 1.00.

Kaplan–Meier analysis demonstrated highly significant overall group differences (log-rank χ^2^ = 58.5; df = 4; *p* = 6 × 10^−12^; Figure 5). Median recurrence-free survival was 10.9 months (PFMT+LAP) and 10.6 months (PFMT+COC), compared with 9.2 months (PFMT), 9.0 months (LAP-only), and 9.0 months (COC-only).

Multivariable Cox regression (Table 4) confirmed significantly lower recurrence hazards for PFMT+LAP (HR = 0.384; 95% CI 0.266–0.555; *p* < 0.001) and PFMT+COC (HR = 0.408; 95% CI 0.279–0.597; *p* < 0.001) vs. LAP-only. COC-only was not significantly different (HR = 1.11; *p* = 0.559). Global Schoenfeld test *p* = 0.463 confirmed proportional hazards assumption.

**TABLE 4:** Multivariable Cox proportional hazards regression (reference: LAP-only; *n* = 446).

Group	HR	95% CI	*p*
PFMT	0.731	0.516–1.04	0.079
PFMT+LAP	0.384	0.266–0.555	<0.001
PFMT+COC	0.408	0.279–0.597	<0.001
COC-only	1.110	0.788–1.55	0.559

HR, hazard ratio; CI, confidence interval. Adjusted for 28 baseline covariates. Schoenfeld global *p* = 0.463.

### 3.8 Adverse events

Adverse-event profiles differed notably across the five study groups (Table 5). No irregular bleeding events were recorded among patients receiving PFMT alone, and none discontinued treatment because of adverse events. For patients allocated to PFMT + LAP, irregular bleeding occurred in 50.5 % of participants, mean weight change was 2.58 ± 1.28 kg, and 4.4 % discontinued therapy owing to adverse events. The PFMT + COC group demonstrated a 10.3 % rate of irregular bleeding, a mean weight change of 0.61 ± 1.02 kg, with 8.0 % treatment discontinuation secondary to adverse events. In the LAP-only arm, irregular bleeding affected 57.6 % of patients, mean weight gain reached 3.31 ± 1.54 kg, and 7.1 % stopped treatment due to adverse events. Among COC-only patients, irregular bleeding was observed in 18.8 %, mean weight change was 0.54 ± 1.07 kg, and 11.8 % discontinued treatment because of adverse events. Group-level comparisons revealed highly statistically significant differences for irregular bleeding (*x*^2^ = 117.74) and weight change (*F* = 129.9), both (*p* < 2×10^−16^).

**TABLE 5:** Adverse event summary across five treatment groups.

Group	*n*	Irregular bleeding [*n* (%)]	Weight change (mean ± SD, kg)	Discontinued due to AE [*n* (%)]
PFMT	98	0 (0%)	0.17 ± 0.81	0 (0%)
PFMT+LAP	91	46 (50.5%)	2.58 ± 1.28	4 (4.4%)
PFMT+COC	87	9 (10.3%)	0.61 ± 1.02	7 (8.0%)
LAP-only	85	49 (57.6%)	3.31 ± 1.54	6 (7.1%)
COC-only	85	16 (18.8%)	0.54 ± 1.07	10 (11.8%)

AE, adverse event. Irregular bleeding χ^2^ = 117.74; weight change *F* = 129.9; both *p* < 2 × 10^−16^.

## 4 Discussion

Endometriosis-associated pain is multifactorial, rooted in chronic inflammation, neuroangiogenesis, central and peripheral sensitization, and maladaptive pelvic floor muscle function (23). Surgical removal of endometriotic lesions does not necessarily resolve the underlying disease process, as evidenced by high recurrence rates in long-term follow-up studies of postoperative hormonal regimens (24). Residual micro-implants, ongoing inflammation, and visceromotor hyperalgesia can perpetuate symptomatic recurrence despite apparently successful surgery (25). This necessitates postoperative adjuvant therapies capable of simultaneously suppressing endometrial tissue activity and addressing musculoskeletal contributors (26).

Pelvic floor muscle training alone has demonstrated value for patients with chronic pelvic pain and dyspareunia, as muscle dysfunction and hypertonicity frequently compound the pain experience in endometriosis (27). Physical rehabilitation enhances muscular coordination, reduces pelvic muscle spasm, and may facilitate the downregulation of central pain pathways by modulating afferent input (28). Randomized controlled trial evidence further supports the efficacy of PFMT in improving pelvic floor function and alleviating pain in women with deep infiltrating endometriosis (29). However, the evidence base for PFMT in endometriosis remains heterogeneous: a recent cross-sectional survey of self-reported outcomes found little to no benefit from pelvic floor physical therapy in a substantial proportion of patients, with some reporting symptom worsening irrespective of surgical history (30). Unlike that survey, which relied on retrospective patient recall without a comparator group or objective outcome measures, the present study applied propensity score matching against dedicated hormone-only controls with objective 12-month recurrence and survival endpoints, which may partly explain the more favorable findings reported here. The present study confirms that even PFMT alone reduces dysmenorrhea recurrence relative to hormone-only controls (OR = 0.518; *p* = 0.048), suggesting an independent neuromuscular analgesic effect (10).

The addition of LAP or COC amplifies these benefits through complementary mechanisms. Progestogens induce amenorrhoea and sustained suppression of endometrial proliferation by downregulating gonadotropin secretion and antagonizing estrogen-receptor-mediated cellular proliferation within residual ectopic tissue (31, 32). Agents targeting the hypothalamic-pituitary-ovarian axis, including GnRH antagonists, have further demonstrated that sustained inhibition of this signaling cascade disrupts the neurovascular infiltration and cytokine-mediated pain sensitization that perpetuate endometriotic lesions (33). The genetic underpinnings of endometriosis, including susceptibility loci governing hormonal signaling, may partly explain why individual patients respond differentially to hormonal suppression (34). Combined oral contraceptives suppress ovulation and stabilize the hormonal milieu, while both COC and progestogens are associated with upregulation of anti-nociceptive pathways that may blunt central sensitization (35). Environmental and endocrine factors that destabilize the systemic hormonal environment may conversely perpetuate lesion reactivation, further underscoring the value of sustained pharmacological suppression (36).

The synergy observed when combining PFMT with hormonal interventions likely stems from the simultaneous targeting of distinct yet intertwining contributors to pain. Non-pharmacological interventions targeting central sensitization pathways – including acupuncture, which has demonstrated analgesic efficacy through modulation of afferent pain signals in EMS (37) – support the hypothesis that PFMT acts via similar neuromuscular mechanisms. By reducing lesion activity and associated inflammation through hormonal therapy, the pelvic floor muscles may become less reactive, enabling more effective neuromuscular re-education; conversely, improving pelvic floor dynamics may lower neuropathic pain thresholds, rendering patients more responsive to hormonal suppression (38). At the neuromuscular level, chronic pelvic pain in endometriosis is frequently accompanied by pelvic floor hypertonicity and myofascial trigger points, which sustain peripheral nociceptive input and contribute to central sensitization (39). A recent clinical assessment confirmed a high prevalence of pelvic floor trigger points, increased resting muscle tone, and impaired voluntary relaxation in women with endometriosis and chronic pelvic pain, with trigger points in the obturator internus muscle independently associated with poorer pelvic floor and sexual function (40). PFMT protocols that incorporate proprioceptive re-education and graded muscle relaxation, rather than strengthening alone, may interrupt this pain-guarding cycle by reducing resting muscle tone and normalizing afferent signaling. This mechanism is conceptually distinct from, but complementary to, the anti-proliferative and anti-inflammatory actions of hormonal suppression, providing a rationale for combined rather than sequential treatment.

Regarding disease stage, patients with less advanced disease may have a smaller residual endometriotic burden postoperatively, rendering hormonal suppression more capable of eradicating subclinical foci (41). In contrast, advanced-stage endometriosis is characterized by greater tissue infiltration, widespread neuroangiogenesis, and pronounced fibrotic change, making lesions less susceptible to hormonal manipulation and myofascial sequelae more difficult to reverse (42).

The clinically meaningful reduction in NSAID dependence (from 33.67% to approximately 20% in combination groups at 6 months) carries relevance beyond symptom control. An integrated programme combining physical activity, pelvic rehabilitation, and hormonal suppression may collectively reduce analgesic requirements, improving long-term safety in young women for whom chronic NSAID use carries gastrointestinal, renal, and cardiovascular risks (43). Beyond NSAIDs, multimodal pain management in endometriosis may also incorporate neuromodulatory agents, cognitive-behavioral approaches, and complementary modalities such as acupuncture (37); the present findings suggest that PFMT could occupy a comparable role within such a multimodal framework, a hypothesis warranting direct comparison in future prospective studies.

The present study demonstrates convergent evidence from three independent analytic approaches – PSM, multivariable regression, and Kaplan–Meier survival analysis – that PFMT confers significant, reproducible reductions in 12-month pain recurrence beyond hormonal suppression alone. The equivalence of LAP-only and COC-only groups across all outcomes confirms that PFMT, rather than the specific hormonal agent, is the active incremental component.

Several limitations warrant acknowledgement. Most importantly, in the absence of a randomized, placebo-controlled design, the present findings should be regarded as hypothesis-generating evidence of association rather than definitive proof of a causal, clinically actionable benefit; on their own, they are insufficient to support a broad recommendation for PFMT in routine practice. First, the retrospective single-center design introduces selection bias despite PSM with 28 covariates; residual confounding from unmeasured variables cannot be excluded. Second, PSM matched cohort sizes yielded post-hoc power of 0.773–0.816, adequate for the observed large effect sizes but potentially insufficient for smaller secondary outcomes, for example, the PFMT-alone group did not reach statistical significance for CPP recurrence (OR = 0.751, 95% CI 0.391–1.440, *p* = 0.386) or dyspareunia recurrence (OR = 0.699, 95% CI 0.356–1.370, *p* = 0.296), and the corresponding Cox model showed a similarly imprecise estimate for recurrence-free survival (HR = 0.731, 95% CI 0.516–1.04, *p* = 0.079). These comparisons may be more vulnerable to inadequate power than the combination-therapy comparisons, which showed narrower intervals and stronger significance. Third, the 12-month follow-up does not capture the late recurrence profile characteristic of endometriosis, which has been reported to continue rising beyond 24 months postoperatively. Fourth, although between-group differences in VAS dysmenorrhea scores reached statistical significance (β = −0.463 to −0.539 on a 0–10 cm scale, equivalent to approximately 4.6–5.4 mm on a 100 mm VAS), this magnitude is below the empirically validated minimal clinically important difference for endometriosis-associated pelvic pain measured on a VAS (≈10 mm) (44); the clinical significance of the VAS findings should therefore be interpreted more cautiously than the binary recurrence and EHP-30 outcomes, which are less susceptible to this concern. Prospective randomized controlled trials with longer follow-up are needed to confirm these findings.

## 5 Conclusion

In women undergoing surgery for endometriosis, the addition of PFMT to either long-term progestogen or combined oral contraceptives was associated with significant and independent reductions in 12-month pain recurrence across all symptom domains, superior EHP-30 quality-of-life trajectories, reduced NSAID dependence, and superior recurrence-free survival, beyond what hormonal therapy alone achieves. The equivalence of the two hormone-only control groups and the consistent superiority of both combination therapy groups support PFMT as the active incremental component of this association. Given the retrospective design and the absence of a randomized comparator, these findings should be regarded as hypothesis-generating. They provide a rationale for evaluating structured PFMT as an adjunct to postoperative hormonal management for endometriosis, and highlight the need for prospective, randomized, multicenter trials to confirm efficacy and to define optimal protocol intensity, duration, and patient selection criteria.

## Data Availability

The original contributions presented in this study are included in the article/supplementary material, further inquiries can be directed to the corresponding author.
